# Resistance to Recombinant Human Erythropoietin Therapy in a Rat Model of Chronic Kidney Disease Associated Anemia

**DOI:** 10.3390/ijms17010028

**Published:** 2015-12-25

**Authors:** Patrícia Garrido, Sandra Ribeiro, João Fernandes, Helena Vala, Petronila Rocha-Pereira, Elsa Bronze-da-Rocha, Luís Belo, Elísio Costa, Alice Santos-Silva, Flávio Reis

**Affiliations:** 1 Laboratory of Pharmacology & Experimental Therapeutics, Institute for Biomedical Imaging and Life Sciences (IBILI), Faculty of Medicine, University of Coimbra, 3000-548 Coimbra, Portugal; apatricia.garrido@gmail.com (P.G.); fernandesjcg@gmail.com (J.F.); 2 UCIBIO@REQUIMTE, Faculty of Pharmacy, Department of Biological Sciences, Laboratory of Biochemistry, University of Porto, 4050-313 Porto, Portugal; sandra.ribeiro870@gmail.com (S.R.); elsa.rocha@ff.up.pt (E.B.-R.); luisbelo@ff.up.pt (L.B.); emcosta@ff.up.pt (E.C.); assilva@ff.up.pt (A.S.-S.); 3 Center for Studies in Education, and Health Technologies, CI&DETS, CITAB, Agrarian School of Viseu, Polytechnic Institute of Viseu, 3504-510 Viseu, Portugal; hvala@esav.ipv.pt; 4 Research Centre in Health Sciences, University of Beira Interior, 6201-001 Covilhã, Portugal; petronila@live.com.pt; 5 Center for Neuroscience and Cell Biology—Institute for Biomedical Imaging and Life Sciences (CNC.IBILI) Research Consortium, University of Coimbra, 3000-548 Coimbra, Portugal

**Keywords:** chronic kidney disease, anemia, resistance to rHuEPO therapy, erythropoiesis, iron metabolism, kidney hypoxia, inflammation and fibrosis, remnant kidney rat model

## Abstract

This study aimed to elucidate the mechanisms explaining the persistence of anemia and resistance to recombinant human erythropoietin (rHuEPO) therapy in a rat model of chronic kidney disease (CKD)-associated anemia with formation of anti-rHuEPO antibodies. The remnant kidney rat model of CKD induced by 5/6 nephrectomy was used to test a long-term (nine weeks) high dose of rHuEPO (200 UI/kg bw/week) treatment. Hematological and biochemical parameters were evaluated as well as serum and tissue (kidney, liver and/or duodenum) protein and/or gene expression of mediators of erythropoiesis, iron metabolism and tissue hypoxia, inflammation, and fibrosis. Long-term treatment with a high rHuEPO dose is associated with development of resistance to therapy as a result of antibodies formation. In this condition, serum EPO levels are not deficient and iron availability is recovered by increased duodenal absorption. However, erythropoiesis is not stimulated, and the resistance to endogenous EPO effect and to rHuEPO therapy results from the development of a hypoxic, inflammatory and fibrotic milieu in the kidney tissue. This study provides new insights that could be important to ameliorate the current therapeutic strategies used to treat patients with CKD-associated anemia, in particular those that become resistant to rHuEPO therapy.

## 1. Introduction

Chronic kidney disease (CKD) is a debilitating disease affecting about 7% of people over the age of 30, which translates to more than 70 million people in developed countries worldwide [1]. The increased prevalence of diabetes, hypertension and obesity, as well as the aging of the population, decisively contribute to perpetuate the rise of CKD [2,3]. Several studies have documented that patients with CKD are at higher risk of cardiovascular diseases than the general population, and show a higher rate of cardiovascular mortality, particularly end-stage renal disease (ESRD) patients, who have a 500-fold greater risk than age-matched controls with normal renal function [4,5,6,7].

Anemia, a very common major complication of CKD, is already observed in early stages of CKD (stage 2) and its prevalence and severity increases as renal failure progresses to more advanced stages [8]. The most well-known cause of CKD anemia is an inadequate erythropoietin (EPO) production; however, several events associated with the disease, including chronic inflammation, blood loss, vitamin deficiencies, decreased iron absorption and utilization, might contribute also to the anemia of CKD [8,9]. Recombinant human erythropoietin (rHuEPO) therapy has been used to correct CKD associated-anemia, particularly in ESRD patients, improving their quality of life [9,10,11,12,13,14]. However, the impact on morbidity and mortality remains debatable, mainly due to the potential for increase in adverse cardiovascular effects, namely increased risk of stroke, venous thromboembolism and mortality [15,16,17]. There is a marked variability in the sensitivity to rHuEPO, with up to 10-fold changeability in dose requirements to achieve correction of the anemia, and 5%–10% of CKD patients show weak responses [18]; this hyporesponsiveness (or resistance) to rHuEPO therapy is associated with higher morbidity and mortality in ESRD patients [19,20]. Although the mechanisms underlying this variability in response are unclear [21,22], resistance to rHuEPO therapy has been associated with inflammation, oxidative stress and iron deficiency, as major causes, and with blood loss, hyperparathyroidism, aluminum toxicity and vitamin B12 or folate deficiencies, as minor causes [21,22,23,24]. In addition, a serious adverse effect of the long-term rHuEPO treatment is pure red cell aplasia (PRCA). Although rHuEPO is weakly immunogenic, it may induce the production of immunoglobulin (Ig) G antibodies against the recombinant molecules and the residual endogenous EPO [25,26,27]. This adverse effect might become more common with the introduction of biosimilar products, but the mechanisms underlying this form of resistance and the impact on the typical features of this anemia remains to be elucidated.

Recent evidences suggest that CKD anemia might be due to a defective hypoxic signaling rather than an inability of the EPO-producing cells to synthesize EPO [28,29]. A disturbance in iron homeostasis is also a hallmark of the anemia of CKD, which usually presents as a functional iron deficient anemia, with low serum iron and transferrin alongside with normal or even high ferritin [30] explained by the underlying inflammatory process in CKD patients, presenting increased hepcidin levels [31]. Hepcidin controls enterocyte iron absorption and macrophage iron mobilization by linking to the iron exporter ferroportin present on the surface of those iron-releasing cells, triggering its degradation [31]. In a previous study, we were able to describe iron metabolism, kidney hypoxia, inflammation and fibrosis features in a rat model of CKD-associated anemia [29].

In addition, we have recently showed that a long-term (nine weeks) treatment of rats with a high dose of rHuEPO (200 IU/kg bw/week) leads to anti-rHuEPO antibodies formation [32]. In this rat model of antibody-mediated erythroid hypoplasia without CKD, we found several changes in erythropoiesis and iron metabolism. The anti-rHuEPO antibodies inhibited both endogenous and recombinant EPO, leading to the development of anemia. This anemia leads to a serum iron increase, stimulating hepcidin expression, despite no evidence of inflammation; thus, iron seems to be the key modulator of hepcidin synthesis under these circumstances. The aim of this study is to clarify the mechanisms underlying this hyporesponse to endogenous EPO, as well as the impact of the long-term treatment of anemia with high doses of rHuEPO associated with antibody mediated erythroid hypoplasia, in a rat model of CKD induced by 5/6 nephrectomy previously characterized by us [29], focusing on iron metabolism, kidney hypoxia, inflammation and fibrosis.

## 2. Results

### 2.1. Body and Tissue Weights and Blood Pressure

The CRF rats presented significantly reduced (*p* < 0.05) BW and higher (*p* < 0.01) KW/BW and HW/BW ratios, when compared with Sham group, although KW and HW were similar (Table 1). The CRF rats treated with 200 IU rHuEPO exhibited values similar to those found for CRF animals, excepting a significantly lower (*p* < 0.05) KW/BW.

**Table 1:** Body and tissue weights, blood pressure and heart rate, hematological and biochemical data, at the end of protocol.

Parameters	Sham	CRF	CRF + 200 IU rHuEPO
*Body and tissues weights*			
BW (kg)	0.45 ± 0.02	0.36 ± 0.01 ^a,1^	0.39 ± 0.01
KW (g)	1.22 ± 0.03	1.65 ± 0.04	1.69 ± 0.08 ^a^
KW/BW (g/kg)	2.72 ± 0.05	4.61 ± 0.22 ^aa^	4.34 ± 0.20 ^aa,b^
HW (g)	1.16 ± 0.03	1.24 ± 0.07	1.25 ± 0.04
HW/BW (g/kg)	2.58 ± 0.08	3.48 ± 0.25 ^aa^	3.21 ± 0.14 ^a^
LW (g/kg)	13.33 ± 0.48	11.32 ± 0.34	13.44 ± 0.42
LW/BW (g/kg)	29.61 ± 0.65	31.43 ± 0.71	34.41 ± 1.14 ^a^
*Blood pressure and heart rate*			
SBP (mmHg)	117.7 ± 1.15	134.1 ± 4.6 ^aa^	169.1 ± 1.5 ^aaa,bbb^
DBP (mmHg)	110.1 ± 0.59	113.2 ± 2.08	133.3 ± 7.2 ^aaa,bb^
MBP (mmHg)	115.0 ± 0.97	117.5 ± 1.21	143.9 ± 4.8 ^aaa,bbb^
HR (beats/min)	357.7 ± 2.74	367.6 ± 5.19	376.4 ± 3.2
*Hematological data*			
WBC (×10^3^/µL)	1.78 ± 0.30	5.01 ± 1.76	3.16 ± 0.70
MCV (fL)	52.52 ± 0.53	51.93 ± 0.69	52.83 ± 0.81
MCH (pg)	18.08 ± 0.18	18.36 ± 0.24	18.60 ± 0.19
MCHC (g/dL)	34.60 ± 0.08	35.37 ± 0.19 ^aa^	35.26 ± 0.30
RDW (%)	11.48 ± 2.53	18.34 ± 3.23	17.88 ± 0.67 ^a,b^
PLT (×10^3^/µL)	713.75 ± 15.19	769.00 ± 73.17	786.00 ± 50.83
PDW (%)	16.34 ± 0.18	16.44 ± 0.20	16.44 ± 0.24
*Biochemical parameters*
TGs (mmol/L)	1.05 ± 0.14	1.58 ± 0.32	1.58 ± 0.29
Total-c (mmol/L)	1.25 ± 0.06	2.44 ± 0.54 ^a^	2.74 ± 0.25
CK (U/L)	540.57 ± 58.94	473.00 ± 85.57	294.86 ± 35.86 ^a,b^
ALT (U/L)	35.17 ± 2.21	42.00 ± 18.53 ^a^	26.14 ± 1.18 ^a^
AST (U/L)	80.57 ± 7.84	139.43 ± 70.70	54.57 ± 3.02 ^a,b^
Bilirubin (µmol/L)	8.04 × 10^−5^ ± 1.03 × 10^−5^	1.03 × 10^−5^ ± 1.71 × 10^−5^	1.20 × 10^−5^ ± 1.71 × 10^−5^
IL-6 (pg/mL)	132.29 ± 4.28	138.33 ± 4.22	138.73 ± 1.70
hsCRP (µg/mL)	262.25 ± 12.43	225.31 ± 7.95 ^a^	244.23 ± 7.99
INF-γ (pg/mL)	23.30 ± 3.10	25.51 ± 2.26	25.51 ± 1.22
TGF-β1 (ng/mL)	75.74 ± 5.62	84.13 ± 3.85	72.37 ± 4.55
VEGF (pg/mL)	4.23 ± 0.94	14.16 ± 2.24 ^a^	10.03 ± 1.00 ^a^

^1^ Results are presented as mean ± SEM (7 rats per group): ^a^: *p* < 0.05, ^aa^: *p* < 0.01, and ^aaa^: *p* < 0.001 *vs.* Sham; ^b^: *p* < 0.05, ^bb^: *p* < 0.01, and ^bbb^: *p* < 0.001 *vs.* CRF. ALT: alanine transaminase; AST: aspartate transaminase; BW: body weight; CK: creatine kinase; DBP: diastolic blood pressure; hsCRP: high-sensitive C reactive protein; HR: heart rate; HW: heart weight; KW: kidney weight; LW: liver weight; MBP: mean blood pressure; MCH: mean corpuscular hemoglobin; MCHC: mean cell hemoglobin concentration; MCV: mean corpuscular volume; PDW: platelet distribution width; PLT: platelets; RDW: RBC distribution width; SBP: systolic blood pressure; Total-c: serum total cholesterol; TGs: triglycerides; WBC: white blood cells.

The CRF rats presented significantly increased (*p* < 0.01) SBP and similar DBP, MBP and HR, when compared with the normotensive Sham rats. The CRF rats treated with 200 IU rHuEPO, presented significantly higher values of SBP (*p* < 0.001), DBP (*p* < 0.01) and MBP (*p* < 0.001) when compared with CRF rats (Table 1).

### 2.2. Hematological and Biochemical Data

**Figure 1:**
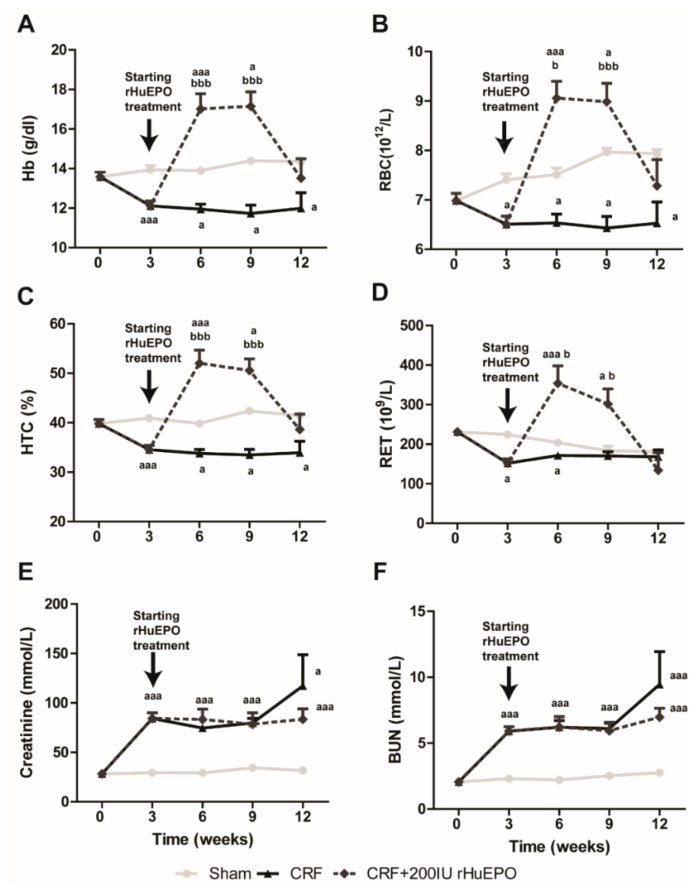
Hematological and renal data throughout the follow-up period of 12 weeks: hemoglobin concentration (**A**); red blood cell count (**B**); hematocrit (**C**); reticulocyte count (**D**); creatinine (**E**); and BUN concentrations (**F**). Results are presented as mean ± SEM (seven rats per group): a: *p* < 0.05 and aaa: *p* < 0.001 *vs.* Sham; b: *p* < 0.05 and and bbb: *p* < 0.001 *vs.* CRF.

The hematological and biochemical data for the different groups are presented in Figure 1 and Table 1. The Sham rats showed normal sustained hematologic values throughout the entire study (Figure 1A–D). Three weeks after the 5/6 nephrectomy, the CRF rats developed anemia, as shown by the reduced Hb concentration, RBC count and HTC (*p* < 0.001 for all), when compared to Sham group; RET count presented also a decrease (*p* < 0.05); this anemia persisted until the end of the protocol. In the CRF rats treated with 200 IU rHuEPO therapy, the anemia was corrected and the rats presented significantly increased Hb concentration, HTC, RBC and RET counts, when compared to CRF rats. This erythropoietic response remained until the 9th week, after which the values returned to basal levels (Figure 1A,D).

Concerning the other hematological measures, similar values (WBC, MCV, MCH, PLT and PDW) were found between CRF and Sham group, excepting an increased (*p* < 0.01) MCHC in the CRF rats. No significant differences were found between CRF, Sham and rHuEPO-treated rats, excepting for a reduced (*p* < 0.05) RDW in Sham rats (Table 1).

In CRF rats, significantly (*p* < 0.001) increased serum creatinine and BUN concentrations were found three weeks after 5/6 nephrectomy, as compared to Sham (Figure 1E,F, respectively). These values remained high until the ninth week, after which a further increase was observed at the final time (*p* < 0.001 and *p* < 0.05, respectively), when compared with Sham group. Similar values of serum creatinine and BUN were observed between CRF rats treated with rHuEPO and those without treatment, throughout the entire protocol. Regarding the other biochemical data, the CRF rats presented increased (*p* < 0.05) total-cholesterol, ALT and VEGF, when compared with Sham rats (Table 1). All the analyzed parameters were similar between untreated and rHuEPO treated CRF rats, excepting for a reduced AST in the group under 200 IU rHuEPO treatment.

Serum samples from all animals were also analyzed for anti-rHuEPO antibodies. These antibodies were detected in seven out of seven (100%) CRF rats treated with 200 IU rHuEPO, presenting a title of 1:2, while in all the Sham and CRF rats the anti-rHuEPO antibodies were undetected.

### 2.3. Serum EPO Concentration and EPO and EPOR mRNA Expression in the Liver and Kidney

**Figure 2:**
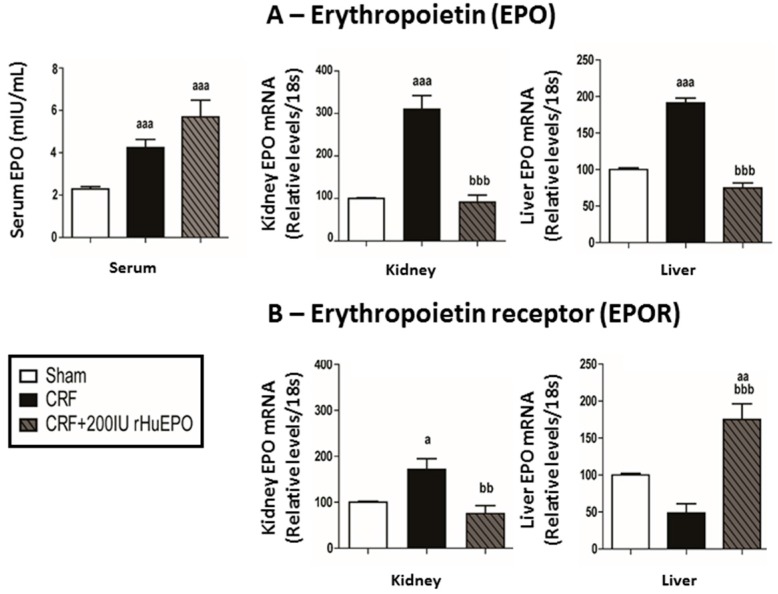
Erythropoietin (EPO) and erythropoietin receptor (EPOR): EPO in serum and kidney and liver mRNA levels (**A**); and EPOR kidney and liver mRNA levels (**B**), at the end of the study (12 weeks). Results are presented as mean ± SEM (seven rats per group): a: *p* < 0.05; aa: *p* < 0.01; and aaa: *p* < 0.001 *vs.* Sham; bb: *p* < 0.01; and bbb: *p* < 0.001 *vs.* CRF.

Both CRF untreated and rHuEPO-treated rats showed significantly higher (*p* < 0.001) serum endogenous EPO concentrations, when compared with Sham group (Figure 2A). There was a notable (*p* < 0.05) overexpression of EPO and EPO receptor (EPOR) mRNA in the kidney tissue of CRF rats, when compared with Sham rats, an effect that was abolished in the presence of rHuEPO treatment (Figure 2A,B). In the liver tissue, a significant overexpression (*p* < 0.001) of EPO mRNA was found in CRF rats, when compared with Sham, which was not observed in rats under rHuEPO treatment. EPOR mRNA levels were similar for all groups, excepting for a significant (*p* < 0.001) overexpression in CRF rats treated with 200 IU rHuEPO (Figure 2A,B).

### 2.4. Iron Metabolism

**Figure 3:**
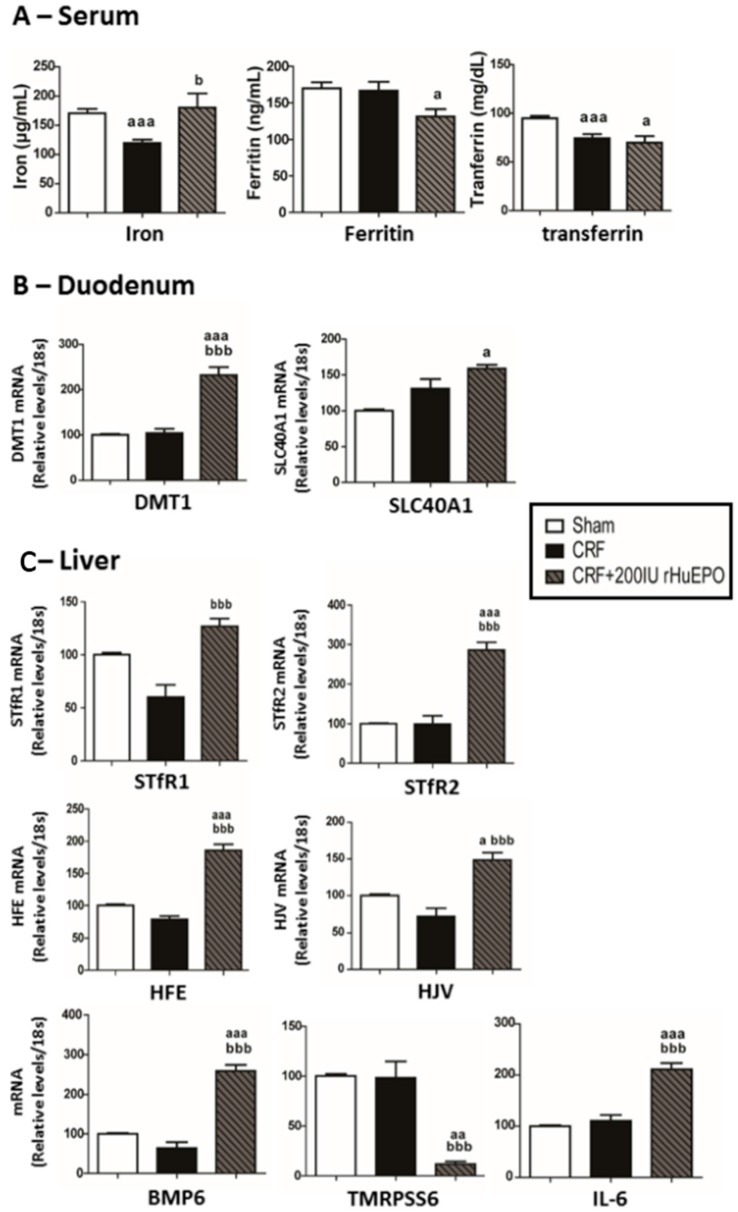
Iron metabolism: Serum iron, ferritin and transferrin at final time (**A**); Relative gene expression mRNA levels/18S of DMT1 and SLC40A1 in the duodenum (**B**); and of iron regulatory proteins in the liver (**C**) at the end of protocol (12 weeks). Results are presented as mean ± SEM (7 rats per group): a: *p* < 0.05; aa: *p* < 0.01; and aaa: *p* < 0.001 *vs.* Sham; b: *p* < 0.05; and bbb: *p* < 0.001 *vs.* CRF. Ferroportin (*SLC40A1*), hemojuvelin (*HJV*), soluble transferrin receptor (*STFR*), hemochromatosis (*Hfe*), divalent metal transporter 1 (*DMT1*), transferrin receptor 1 (*TfR1*), matriptase-2 (*TMPRSS6*), interleukin-6 (*IL-6*) and bone morphogenic protein 6 (*BMP6*).

The CRF rats, as compared to Sham, showed a significant (*p* < 0.001) decrease in serum iron and transferrin, and similar ferritin levels. The 200 IU rHuEPO-treated CRF rats presented iron, transferrin and ferritin serum levels similar to those of Sham rats; when compared to CRF rats, a significant (*p* < 0.05) increase in serum iron and no changes in ferritin and transferrin serum levels were found (Figure 3A). In the duodenum, no significant differences were observed for SLC40A1 and DMT1 mRNA expression between CRF and Sham rats. However, in the CRF + 200 IU rHuEPO group, there was a significant (*p* < 0.001) overexpression of DMT1 and a trend (*p* = 0.074) to increased expression of SLC40A1, when compared with the CRF group (Figure 3B).

**Figure 4:**
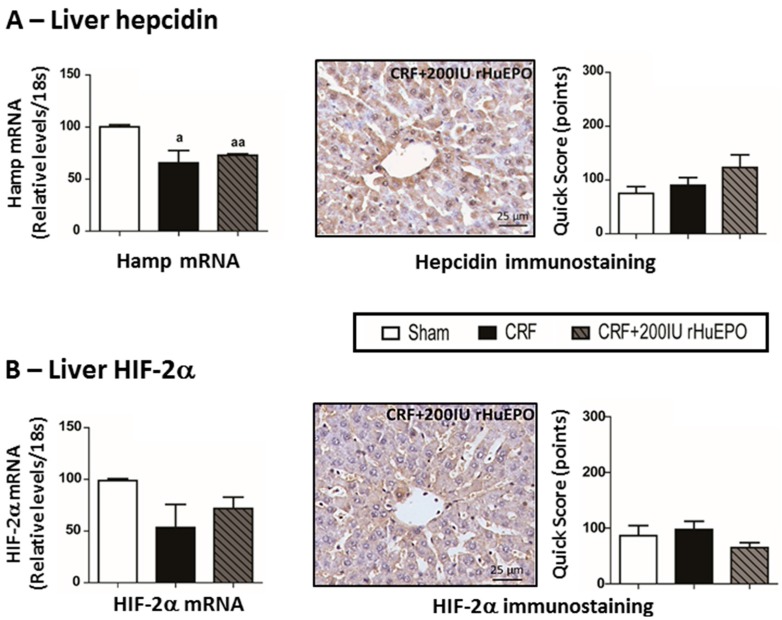
Liver hepcidin (**A**); and HIF2α (**B**) expression of mRNA and protein (immunohistochemical staining). Original magnification ×400. Results are presented as mean ± SEM (seven rats per group): a: *p* < 0.05; and aa: *p* < 0.01 *vs.* Sham.

In the liver tissue, no significant alterations were found for mRNA expression of several iron regulatory proteins (*sTfR1*, *sTfR2*, *Hfe*, *HJV*, *BMP6*, *TMPRSS6*, *IL-6* and *HIF-2α*) in the CRF group when compared with the Sham one (Figure 3C). However, there was a significantly reduced (*p* < 0.05) expression of Hamp mRNA in the CRF rats, when compared with Sham animals (Figure 4A). The CRF rats under 200 IU rHuEPO therapy, as compared with the CRF ones, showed significant changes in the liver expression of most of those mediators of iron metabolism, namely a significant (*p* < 0.001) overexpression of *sTfR1*, *sTfR2*, *Hfe*, *HJV*, *BMP6* and *IL-6*, and a significantly reduced expression of *TMPRSS6*, *Hamp* and *HIF-2α* mRNA in the liver presented similar values *vs.* CRF rats (Figure 3C and Figure 4A,B, respectively). In addition, no significant differences were observed between groups for hepcidin and HIF-2α protein expression (immunostaining) in the liver tissue (Figure 4A,B, respectively).

### 2.5. Kidney Lesions

**Figure 5:**
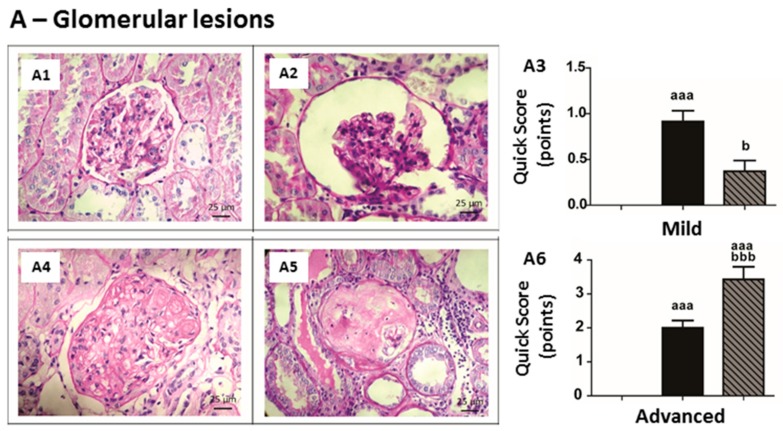

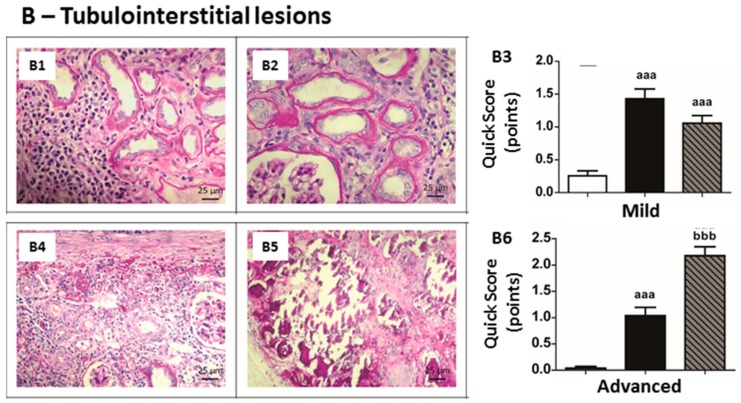
Kidney histopathology. Representative glomerular and tubulointerstitial lesions observed in kidneys of rat groups under study, at the final time (PAS staining, original magnification ×400). (**A1**) glomerular hypercellularity; (**A2**) dilatation of the Bowman’s space and glomerular atrophy; (**A3**) total score of mild glomerular lesions for each rat group; (**A4**) nodular sclerosis; (**A5**) global glomerulosclerosis; (**A6**) total score of advanced glomerular lesions for each rat group; (**B1**) interstitial inflammatory infiltration; (**B2**) tubular basements membrane irregularity; (**B3**) total score of mild tubulointerstitial lesions in lesions for each rat group; (**B4**) Interstitial fibrosis and tubular atrophy (IFTA); (**B5**) tubular calcification; and (**B6**) total score of advanced tubulointerstitial lesions for each rat group. Results are presented as mean ± SEM (seven rats per group): aaa: *p* < 0.001 *vs.* Sham; b: *p* < 0.05, and bbb: *p* < 0.001 *vs.* CRF.

No significant changes were found in kidney histomorphology of Sham rats after the experimental period (Table 2 and Table 3; Figure 5). However, CRF rats presented several glomerular and tubulointerstitial lesions. Concerning mild glomerular lesions, most of the CRF rats presented thickening of Bowman’s capsule, hyalinosis of vascular pole, glomerular atrophy and hypercellularity (Table 2; Figure 5A). In addition, all CRF rats presented at least one of the advanced glomerular lesions, and mesangial expansion was present in five out of seven rats (Table 2; Figure 5A). The CRF + 200 IU rHuEPO rats, as compared to CRF ones, displayed an improvement in mild glomerular lesions (Figure 5A3); however, the advanced lesions were still with a predominance of global glomerulosclesosis (five out of seven rats) (Table 2; Figure 5A6). The changes reported are viewed as the total scores of mild and advanced glomerular lesions (Table 2 and Figure 5A3,6).

**Table 2:** Scoring and distribution (%) of mild and advanced glomerular kidneys lesions.

Mild Lesions	Group	Score (*n*, %)				
		0 (Absent)	1 (<25%)	2 (25%–50%)	3 (>50%)	Total Score
**Thickening of Bowman´s Capsule**	Sham	7 (100%)	0	0	0	0.00 ± 0.00
	CRF	1 (14.3%)	3 (42.9%)	1 (14.3%)	2 (28.6%)	1.57 ± 0.43 ^a,1^
	CRF + 200 IU rHuEPO	3 (42.9%)	3 (42.9%)	1 (14.3%)	0	0.71 ± 0.28
**Hyalinosis of the Vascular Pole**	Sham	7 (100%)	0	0 (0%)	0 (0%)	0.00 ± 0.00
	CRF	1 (14.3%)	6 (85.7%)	0	0	0.86 ± 0.14
	CRF + 200 IU rHuEPO	5 (71.4%)	1 (14.3%)	0	1 (14.3%)	0.57 ± 0.43
**Glomerular Atrophy**	Sham	7 (100%)	0	0	0	0.00 ± 0.00
	CRF	1 (14.3%)	6 (85.7%)	0	0	0.86 ± 0.14 ^a^
	CRF + 200 IU rHuEPO	5 (71.4%)	2 (28.6%)	0	0	0.29 ± 0.18
**Hypercellularity**	Sham	7 (100%)	0	0	0	0.00 ± 0.00
	CRF	0	7 (100%)	0	0	1.00 ± 0.00 ^aa^
	CRF + 200 IU rHuEPO	7 (100%)	0	0	0	0.00 ± 0.00 ^bb^
**Dilatation of the Bowman’s Space**	Sham	7 (100%)	0	0	0	0.00 ± 0.00
	CRF	5 (71.4%)	2 (28.6%)	0	0	0.29 ± 0.18
	CRF + 200 IU rHuEPO	5 (71.4%)	2 (28.6%)	0	0	0.28 ± 0.18
**Total Group Score**	Sham					0.00 ± 0.00
	CRF					0.91 ± 0.12 ^aaa^
	CRF + 200 IU rHuEPO					0.37 ± 0.12 ^b^
**Group**	**Advanced Glomerular Lesions (*n*, %)**					
	**None of the Previous Lesions (0)**	**Thickening of GBM (1)**	**Mesangial Expansion (2)**	**Nodular Sclerosis (3)**	**Global Glomerulosclerosis (4)**	**Total Group Score**
Sham	7 (100%)	0	0	0	0	0.00 ± 0.00
CRF	0	1 (14.3%)	5 (71.4%)	1 (14.3%)	0	2.00 ± 0.22 ^aaa^
CRF + 200 IU rHuEPO	0	0	2 (28.6%)	0	5 (71.4%)	3.43 ± 0.37 ^aaa,bbb^

^1^ Results are presented as mean ± SEM (seven rats per group): ^a^: *p* < 0.05, ^aa^: *p* < 0.01, and ^aaa^: *p* < 0.001 *vs.* Sham; ^b^: *p* < 0.05, ^bb^: *p* < 0.01, and ^bbb^: *p* < 0.001 *vs.* CRF.

**Table 3:** Scoring and distribution (%) of mild and advanced tubulointerstitial kidney lesions.

**Mild Lesions**	**Group**	**Score (*n*, %)**				
		**0 (Absent)**	**1 (<25%)**	**2 (25%–50%)**	**3 (>50%)**	**Total Score**
**Tubular Hyaline Droplets**	Sham	7 (100%)	0	0	0	0.00 ± 0.00
	CRF	0	7 (100%)	0	0	1.00 ± 0.00 ^aaa^
	CRF + 200 IU rHuEPO	0	7 (100%)	0	0	1.00 ± 0.00 ^aaa^
**TBM Irregularity**	Sham	7 (100%)	0	0	0	0.00 ± 0.00
	CRF	0	3 (42.9%)	2 (28.5%)	2 (28.5%)	1.86 ± 0.34 ^aaa^
	CRF + 200 IU rHuEPO	0	5 (71.4%)	2 (28.5%)	0	1.29 ± 0.18 ^a^
**Tubular Dilatation**	Sham	7 (100%)	0	0	0	0.00 ± 0.00
	CRF	2 (28.5%)	1 (14.3%)	2 (28.5%)	2 (28.5%)	1.57 ± 0.48 ^a^
	CRF + 200 IU rHuEPO	7 (100%)	0	0	0	0.00 ± 0.00 ^bb^
**Interstitial Inflammatory Infiltrate**	Sham	4 (57.2%)	3 (42.9%)	0	0	0.29 ± 0.18
	CRF	0	0	7 (100%)	0	2.00 ± 0.00 ^aaa^
	CRF + 200 IU rHuEPO	0	0	7 (100%)		2.00 ± 0.00 ^aaa^
**Vacuolar Tubular Degeneration**	Sham	0	7 (100%)	0	0	1.00 ± 0.00
	CRF	3 (42.9%)	3 (42.9%)	1 (14.3%)	0	0.71 ± 0.29 ^aaa^
	CRF + 200 IU rHuEPO	0	7 (100%)	0	0	1.00 ± 0.00 ^bb^
**Total Group Score**	Sham					0.26 ± 0.08
	CRF					1.43 ± 0.15 ^aaa^
	CRF + 200 IU rHuEPO					1.06 ± 0.12 ^aaa^
**Advanced Lesions**	**Group**	**Score (*n*, %)**				
		**0 (Absent)**	**1 (<25%)**	**2 (25%–50%)**	**3 (>50%)**	**Total Score**
**Hyaline Cylinders**	Sham	6 (85.7%)	1 (14.3%)	0	0	0.14 ± 0.14
	CRF	0	1 (14.3%)	6 (85.7%)	0	1.86 ± 0.14 ^aa^
	CRF + 200 IU rHuEPO	0	0	4 (57.1%)	3 (42.9%)	2.43 ± 0.20 ^aaa^
**Tubular Calcification**	Sham	7 (100%)	0	0	0	0.00 ± 0.00
	CRF	7 (100%)	0	0	0	0.00 ± 0.00
	CRF + 200 IU rHuEPO	1 (14.3%)	6 (85.7%)	0	0	0.85 ± 0.14 ^aaa,bbb^
**Necrosis**	Sham	7 (100%)	0	0	0	0.00 ± 0.00
	CRF	2 (28.6%)	5 (71.4%)	0	0	0.71 ± 0.18
	CRF + 200 IU rHuEPO	0	0	4 (57.1%)	3 (42.9%)	2.43±0.20 ^aaa,bbb^
**IFTA**	Sham	7 (100%)	0	0	0	0.00 ± 0.00
	CRF	0	3 (42.9%)	4 (57.1%)	0	1.57 ± 0.20 ^aaa^
	CRF + 200 IU rHuEPO	0	0	0	7 (100%)	3.00 ± 0.00 ^aaa,bbb^
**Total Group Score**	Sham					0.04 ± 0.04
	CRF					1.04 ± 0.16 ^aaa^
	CRF + 200 IU rHuEPO					2.18 ± 0.17 ^aaa,bbb^

^1^ Results are presented as mean ± SEM (seven rats per group): ^a^: *p* < 0.05, ^aa^: *p* < 0.01, and ^aaa^: *p* < 0.001 *vs.* Sham; ^bb^: *p* < 0.01, and ^bbb^: *p* < 0.001 *vs.* CRF.

Concerning the mild tubulointerstitial lesions, all animals of the CRF group presented tubular hyaline droplets, TBM irregularity, interstitial inflammatory infiltration, and most of them presented tubular dilatation, as compared to Sham rats (Table 3 and Figure 5B). The group of CRF rats treated with rHuEPO, as compared to CRF rats without treatment, showed a trend (*p* = 0.069) towards a reduction in mild tubulointerstitial lesions (Table 3 and Figure 5B3), but a significant increase in advanced lesions, namely in hyaline cylinders, calcification, necrosis and IFTA (Table 3 and Figure 5B6).

As the changes in serum iron presented by CRF rats and CRF rats treated with rHuEPO could be due to iron leakage due to kidney lesions, we performed iron staining according to Perls method. We found that iron deposits were almost undetectable in Sham rats, increased in CRF rats (present in six out of seven animals) and were prevented in the CRF + 200 IU rHuEPO rats (data not shown).

### 2.6. Mediators of Kidney Lesions

**Figure 6:**
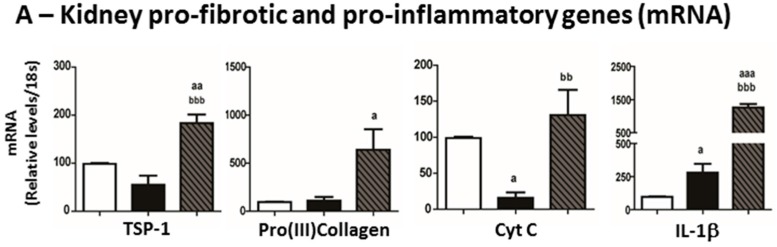

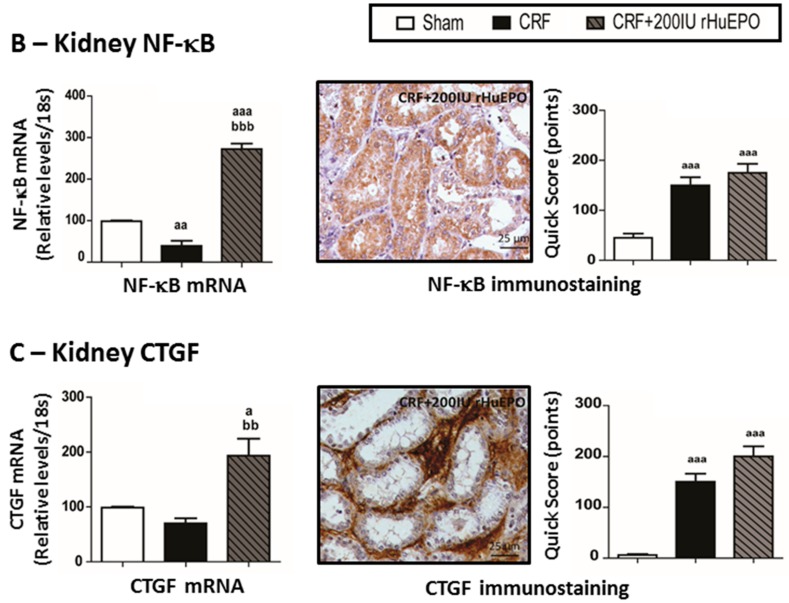
Kidney expression of mediators of inflammation and fibrosis. Gene (mRNA) expression of thrombospondin-1 (TSP-1), pro-(III) collagen, cytochrome c (Cyt C) and interleukin 1β (IL-1β) (**A**); Kidney expression of nuclear factor kappa B (NF-κB) gene and protein (immunohistochemical staining) (**B**) and connective tissue growth factor (CTGF) gene and protein (**C**). Original magnification × 400. Results are presented as mean ± SEM (seven rats per group): a: *p* < 0.05, aa: *p* < 0.01, and aaa: *p* < 0.001 *vs.* Sham; bb: *p* < 0.01, and bbb: *p* < 0.001 *vs.* CRF.

No significant change was observed in kidney mRNA expression of TSP-1, Pro(III) collagen and CTGF in CRF rats, when compared with Sham, however a down-regulation of CytC and NF-κB expression (*p* < 0.05 and *p* < 0.01, respectively) was observed (Figure 6A,B). Major changes in kidney mRNA expression was observed in the CRF + 200 IU rHuEPO rats versus CRF animals; in fact, a significant overexpression of IL-1β, TSP-1, CytC, NF-κB and CTGF were found (Figure 6). Increased protein (immunostaining) expression of NF-κB and CTGF was encountered in the rHuEPO-treated CRF rats when compared with those untreated (Figure 6B,C, respectively).

### 2.7. Kidney mRNA and Protein Expression of Hypoxia Inducible Factor 2α and 2β

**Figure 7:**
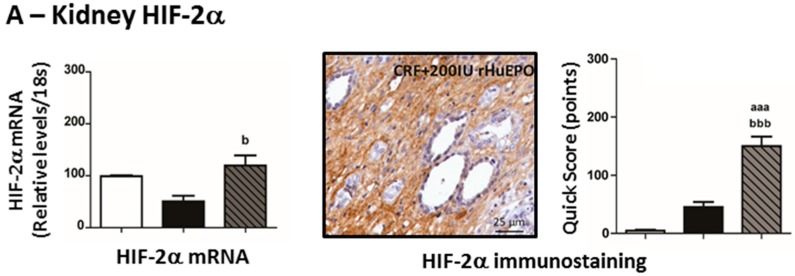

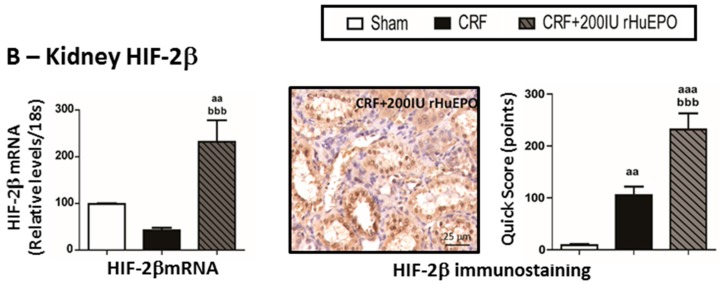
Kidney hypoxia-inducible factor 2α (**A**); and 2β (**B**). mRNA and protein (immunohistochemical staining) expression. Original magnification ×400. Results are presented as mean ± SEM (7 rats per group): aa: *p* < 0.01, and aaa: *p* < 0.001 *vs.* Sham; b: *p* < 0.05, and bbb: *p* < 0.001 *vs.* CRF.

No significant change was observed in kidney mRNA HIF-2α expression in the CRF rats, when compared to Sham animals, although a trend (*p* = 0.074) towards an increased protein expression (immunostaining) was found. The CRF + 200 IU rHuEPO rats presented an overexpression of both mRNA and protein in the kidney tissue (Figure 7A). In addition, increased mRNA and protein HIF-2β expression was found in CRF + 200 IU rHuEPO rats, when compared with CRF rats (Figure 7B).

## 3. Discussion

Animal models have been important tools to study the cellular and molecular changes in CKD, and the remnant kidney model is one of the most used models [33,34,35]. Recently we characterized erythropoiesis and iron metabolism in an animal model of erythroid hypoplasia induced by formation of anti-rHuEPO antibodies in Wistar rats long-term treated with rHuEPO therapy [32]. The present work aimed to study the mechanisms underlying the hyporesponse to endogenous EPO, as well as the impact of long-term treatment of anemia with high rHuEPO doses leading to antibody mediated erythroid hypoplasia, in a rat model of CKD induced by 5/6 nephrectomy previously characterized by us [29]. The CRF rats present persistent anemia, without EPO deficiency, with low serum iron and transferrin levels, although iron storage was normal, and unchanged serum IL-6 and hsCRP levels, showing the absence of systemic inflammation. In addition, despite the reduced expression of hepcidin that favors iron absorption, serum iron was reduced, which might be due to iron loss through the damaged kidney. It was reported that in proteinuric conditions, due to glomerular leakage of transferrin, iron might be released from transferrin in the acid milieu of the tubular lumen [36], leading to iron accumulation in the proximal tubule [36,37,38,39] and worsening of CKD. Another hypothesis for the persistence of the anemia in CRF rats is that an altered activity/function of EPO has occurred, resulting from kidney cell damage, supported by previous reports [40,41]. We must also consider the hypothesis that the endogenous EPO concentration is inadequate to overcome anemia.

The treatment of CRF rats with a high dose of rHuEPO (200 IU/kg bw/week) rapidly corrected the anemia, and the Hb concentration reached significantly higher values until the ninth week, as compared to Sham and CRF rats; afterwards, the hematological measures declined to basal values, similar to those of the Sham group, due to formation of EPO-neutralizing antibodies. This condition has been reported in humans [42,43] and, more recently, has increased due to the introduction of EPO biosimilars to treat anemia in some countries [26,27].

The serum EPO levels in CRF group were significantly increased, as compared to Sham rats, and were similar to EPO levels presented by the CRF rats, though presenting a trend towards increased values. This slight increase might be explained by a compensatory production of EPO by the liver, given the significant overexpression of EPOR mRNA in hepatic tissue. While the liver has a role in EPO production in the fetal age, in the adulthood the main producer of EPO is the kidney [44,45,46]. However, in renal disease conditions the extra-renal tissues, such as the liver, might assume a higher part on the compensatory synthesis of EPO. Despite the striking reduction in RBC count, Hb concentration, HCT and reticulocytes, at the end of the protocol, these values were still similar to those presented by Sham rats; actually, as there were still normal values, CRF treated rats showed a kidney and liver EPO gene expression similar to that presented by Sham rats, in opposition to the anemic CRF rats presenting an overexpression.

Renal failure was accompanied by a compensatory renal hypertrophy and angiogenesis in the CRF rats, as revealed by the increase in KW/BW and serum VEGF levels; a high HW/BW ratio was also found in the CRF group, which might be caused by the supplementary effort of the left ventricle muscle to pump blood in this condition of anemia secondary to renal failure development (Table 1). CKD patients usually present a concomitant rise in systolic blood pressure [47,48,49,50], which was also observed in the CRF rats of our study, explaining the cardiac hypertrophy. When treated with 200 IU rHuEPO, there was an aggravation of hypertension, which is a widely described rHuEPO side-effect [47,48,49,50] that contributes to high morbidity and mortality of CKD patients [28,48].

Interestingly, rHuEPO therapy presented a dual impact on kidney lesions. In fact, while an amelioration of mild glomerular and tubulointerstitial lesions was found, advanced lesions were intensified (Table 2 and Table 3 and Figure 5), suggesting the inability to protect the kidney in advanced stages of CKD. Concomitantly, CRF rats treated with 200 IU rHuEPO also presented significantly higher values in protein and/or gene expression of several mediators of kidney inflammation and fibrosis, namely, Cyt C, IL-1β, and CTGF (Figure 6).

Under hypoxic conditions, HIFs promote the transcription of regenerative factors, such as EPO, GLUT receptor, VEGF and CTGF, among others [51,52,53]. However, some of them (namely VEGF and CTGF) might contribute to worsening of kidney disease by promoting inflammation and fibrosis [53,54]. It is becoming widely accepted that, regardless of the initial cause of renal failure, tubulointerstitial fibrosis is the major cause of disease progression [55,56]. Tubulointerstitial damage is typically associated with accumulation of extracellular matrix, infiltration of inflammatory cells, increased number of interstitial fibroblasts, tubular atrophy and finally loss of peritubular capillaries [57]. Given the close association between hypoxia, EPO synthesis, fibrosis and inflammation, it is of major importance to elucidate how rHuEPO therapy affects the evolution of kidney lesions.

**Figure 8:**
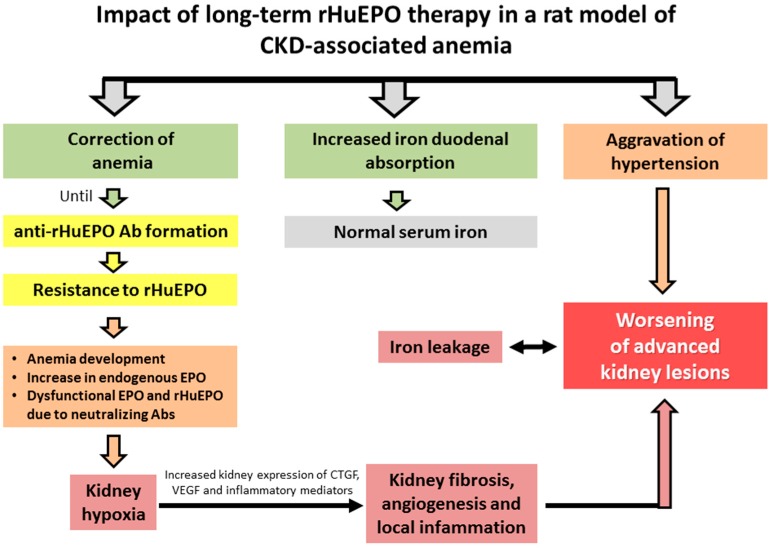
Proposed mechanisms for the impact of resistance to rHuEPO therapy due to anti-EPO antibodies formation in a rat model of CKD-associated anemia.

Despite the still normal Hb concentration, our data is consistent with a hypoxic environment in the kidney of CRF rats treated with rHuEPO, as suggested by the increased expression of HIFs (Figure 7) and by the increase in serum EPO, which is, however, unable to properly stimulate erythropoiesis, due to the formation of EPO-neutralizing antibodies (Figure 8). This is, probably, due to the sudden decrease in Hb concentration leading to a striking reduction in kidney oxygenation; another hypothesis is that the high blood viscosity associated to high RBC concentration during about 3–6 weeks could induce stasis, triggering hypoxia in renal tissue. Hypoxia-induces the expression of local inflammatory and fibrosis mediators, such as NF-κB and CTGF, contributing to aggravation of kidney damage (Figure 8).

Iron metabolism impairment has been viewed as a major feature of CKD-associated anemia and hepcidin (de)regulation is one of the key pieces for resistance to rHuEPO therapy [29,30,58,59,60]. Hepatic Hamp (hepcidin gene) expression is regulated by several proteins, namely Hfe, TfR1, TfR2, HJV, BMP6, matriptase-2 and transferrin [61,62,63]. In the liver, diferric transferrin competes with TfR1 for binding to Hfe, and, when iron is increased, more Hfe is available to bind to TfR2; this complex, TfR2-Hfe, promotes HJV binding to BMP6, increasing hepcidin synthesis [64,65]. Moreover, hypoxia and EPO, as well as twisted gastrulation protein 1 (TWSG1), erythroferrone and growth differentiation factor 15 (GDF15) [66], all produced by erythroblasts, are also important modulators of hepcidin, downregulating its synthesis [66].

In the CRF rats, despite the increased EPO serum levels, anemia persisted and was linked to low serum iron and transferrin levels, while serum IL-6 and hsCRP levels showed the absence of systemic inflammation. The increased expression of duodenal ferroportin favours iron absorption; however, as referred, serum iron is reduced, which might be due to iron leakage in kidney lesions, as showed by tubular iron accumulation (data not shown). In the CRF rats under rHuEPO treatment, iron was normal (similar to Sham rats) and was associated with an overexpression of Tf, TfR2, BMP6, Hfe and HJV in the liver, and, in agreement, with an overexpression of Hamp; moreover, downregulation of matriptase-2 mRNA expression was observed in the liver that might further contribute to overexpression of hepcidin (Figure 3 and Figure 4). However, this hepcidin overexpression was not accompanied by higher protein expression, as showed by the immunochemical studies. A similar profile was found for liver HIF-2α mRNA and protein expression. The normal serum iron levels in the rats treated with rHuEPO, versus untreated, seems to be due to increased iron absorption, as suggested by the duodenal overexpression of iron transporters (DMT1 and ferroportin) (Figure 3). Nevertheless, increased iron availability is not accompanied by recovering of erythropoiesis, which might be due to the formation of anti-EPO antibodies, as well as to the kidney hypoxic, inflammatory and fibrotic milieu, which might be responsible for the impaired EPO erythropoietic activity (Figure 8).

This study has some limitations that future research will cover. One of the aspects for further research is the comparison of our model of CRF-associated anemia induced by 5/6 nephrectomy with other models of anemia, such as the bleeding model used by other groups [67]. This comparison will be important to strength the data regarding the variations of serum EPO and hepcidin levels in the rat, which are strongly influenced by other players, including concentration of Hb, serum iron and ferritin, as previously demonstrated [68,69]. In addition, measure of hepcidin protein levels will be important to complete the information of gene expression. We also acknowledge the interest of evaluating the bone marrow erythropoietic acitivity, by quantification of the myeloid: erythroid ratio. Regarding cardiovascular influences or impact, a longitudinal study of blood pressure data throughout the study time-points will potentially add interesting information to that already observed.

## 4. Experimental Section

### 4.1. Animals and Experimental Protocol

Male Wistar rats (Charles River Lab., Inc., Barcelona, Spain) weighing 300 g were maintained in an air conditioned room, subjected to 12 h dark/light cycles and given standard rat chow (IPM-R20, Letica, Barcelona, Spain) *ad libitum* and free access to tap water. Animal experiments were conducted according to the European Communities Council Directives on Animal Care. The experiments were approved by the Portuguese Foundation for Science and Technology (PTDC/SAU-TOX/114253/2009) and the Local Ethics Committee of the Faculty of Medicine from the University of Coimbra (ORBEA — Organ Responsible for the Animal Welfare).

The rats were divided into three groups (7 rats each): Sham group—submitted to surgical process but without kidney mass reduction and rHuEPO treatment; CRF group—induced by a two-stage (5/6) nephrectomy (about 2/3 of the left kidney was removed by left flank incision and, one week later, the right kidney was removed through identical procedure); and CRF + 200 IU rHuEPO group—treated with rHuEPO (beta epoetin), 200 IU/kg/week s.c., Recormon^®^ (Roche Pharmaceuticals—Roche Farmacêutica Química, Lda., Amadora, Portugal) after the third week of surgery. All animals completed the 12 weeks of protocol. Body weight (BW) was monitored throughout the study and blood pressure (BP) and heart rate (HR) measures were obtained using a tail-cuff sphygmomanometer LE 5001 (Letica, Barcelona, Spain), using the conditions of rat acclimatization and collection of data previously described by us [70].

### 4.2. Sample Collection and Preparation

At the beginning of the experiments (T0) and at 3 (T1), 6 (T2), 9 (T3) and 12 (T4) weeks after the surgical (5/6) nephrectomy, the rats were subjected to intraperitoneal anesthesia with a 2 mg/kg BW of a 2:1 (*v*:*v*) 50 mg/mL ketamine (Ketalar^®^, Parke-Davis, Lab. Pfeizer Lda, Seixal, Portugal) solution in 2.5% chlorpromazine (Largactil^®^, Rhône-Poulenc Rorer, Lab. Vitória, Amadora, Portugal). Blood samples were collected by venipuncuture, from the jugular vein, into vacutainer tubes without anticoagulant (to obtain serum) or with K3EDTA for hematological and biochemical studies; only 3 mL of blood were collected at T0, T1, T2 and T3, to minimize interference with erythropoiesis mechanism and to monitor anemia and renal function; at the end of protocol (T4), 10 mL of blood were collected to perform all the biochemical and hematological assays.

At the end of the protocol, after collection of blood, the rats were sacrificed by cervical dislocation and the kidneys, heart, liver and duodenum were removed, and placed in ice-cold Krebs–Henseleit buffer. The body weight (BW); the weight of kidney (KW) or of the 1/6 remnant kidney; the heart weight (HW); and the liver weight (LW) were measured to calculate the trophism indexes (KW/BW, HW/BW and LW/BW).

### 4.3. Biochemical and Hematological Assays

The following serum markers were analyzed on a Hitachi 717 analyzer (Roche Diagnostics Inc., Massachuasetts, MA, USA) using standard methods: creatinine, blood urea nitrogen (BUN), as renal function markers; glycose, serum total cholesterol (Total-c), triglycerides (TGs), creatine kinase (CK), aspartate transaminase (AST), and alanine transaminase (ALT). Quantification of total bilirubin was performed by a colorimetric test (diazotized sulfanilic acid reaction, Roche Diagnostics). Serum levels of interleukin-6 (IL-6), interferon-γ (IFN-γ), transforming growth factor (TGF-β1) and vascular endothelial growth factor (VEGF) were all measured by rat-specific Quantikine ELISA kits from R&D Systems (Minneapolis, MN, USA). High-sensitive C-reactive protein (hsCRP) was determined by using a rat-specific Elisa kit from Alpha Diagonostic International (San Antonio, TX, USA). Serum levels of erythropoietin (EPO) were evaluated by rat specific ELISA kit (MyBioSource, San Diego, CA, USA). All assays were performed according to the manufacturers’ recommendations.

Red blood cell (RBC) count, hematocrit (Hct), hemoglobin concentration (Hb), mean corpuscular hemoglobin (MCH), mean cell hemoglobin concentration (MCHC), mean corpuscular volume (MCV), RBC distribution width (RDW), platelet distribution width (PDW), platelets (PLT) and white blood cell (WBC) count were assessed in whole blood K3EDTA (Coulter Counter^®^, Beckman Coulter, Inc., Fullerton, CA, USA); reticulocyte (RET) count was performed by microscopic counting on blood smears after vital staining with New methylene blue (Reticulocyte stain, Sigma–Aldrich, St. Louis, MO, USA). Serum iron concentration was determined using a colorimetric method (Iron, Randox Laboratories Ltd., North Ireland, UK), whereas serum ferritin and transferrin were measured by immunoturbidimetry (Laboratories Ltd., North Ireland, UK).

### 4.4. Detection of Anti-EPO Antibodies

To detect anti-EPO antibodies we used an ELISA technique, according to Urra *et al.*, using rHuEPO (Recormon^®^, Roche Pharmaceuticals) as antigen and, as secondary antibody, goat anti-rat IgG conjugated with horseradish peroxidase (Sigma; 100 ng/mL for 1 h, at room temperature) [71]. The substrate tetramethylbenzidine (TMB) (Sigma) was added and the reaction was stopped by the addition of sulfuric acid 1.25 mol/L. The optical density at 450 nm (OD450) was determined with an automatic plate reader.

### 4.5. Gene Expression Analysis

In order to isolate total RNA, 0.2 g samples of liver, duodenum and kidney, from each rat, were immersed in RNA laterTM (Ambion, Austin, TX, USA) upon collection and stored at 4 °C for 24 h; afterwards, samples were frozen at −80 °C. Subsequently, tissue samples weighing 50 ± 10 mg were homogenized in a total volume of 1 mL TRI^®^ Reagent (Sigma, Sintra, Portugal) using a homogenizer, and total RNA was isolated according to TRI^®^ Reagent Kit recommendations. To ensure inactivation of contaminating RNAses, all material used was cleaned and immersed in RNAse-free water (0.2% diethyl pyrocarbonate) for 2 h and finally heated at 120 °C for 1 h. RNA integrity (RIN, RNA Integrity Number) was analyzed using 6000 Nano ChipW kit, in Agilent 2100 bioanalyzer (Agilent Technologies, Walbronn, Germany) and 2100 expert software, following manufacturer instructions. The yield from isolation was from 0.5 to 1.5 μg; RIN values were 7.0–9.0 and purity (A260/A280) was 1.8–2.0. The concentrations of the RNA preparations were confirmed with NanoDrop1000 (ThermoScientific, Wilmington, DE, USA). Possible contaminating remnants of genomic DNA were eliminated by treating these preparations with deoxyribonuclease I (amplification grade) prior to RT-qPCR amplification. Reverse transcription and relative quantification of gene expression were performed as previously described [72]. Real-time qPCR reactions were performed using the primer sequences listed in Table 4 for the genes analyzed. Results were analyzed with SDS 2.1 software (Applied Biosystems, Foster City, CA, USA) and relative quantification calculated using the 2ΔΔ*C*_t_ method [73]. In liver tissue we studied the *EPO*, *EPOR*, *TfR1*, *TfR2*, *Hamp*, *Il-6*, *SLC40A1*, *HJV*, *TF*, *Hfe*, *BMP6* and *TMPRSS6* gene expression; in duodenum tissue the gene expression of DMT1 and SLC40A1 were studied, and in the kidney we evaluated the expression of *EPO*, *EPOR*, *IL-1β*, *TSP-1*, *Pro (III) collagen*, *CytC*, *NF-kB*, *CTGF*, *VEGF*, *HIF-2α* and *HIF-2β* genes.

**Table 4:** List of primer sequences (F: forward; R: reverse).

Gene	Primer Sequences	Gene	Primer Sequences
*EPO*	F: 5’-AGG GTC ACG AAG CCA TGA AG-3’	*IL-6*	F: 5’-CGA GCC CAC CAG GAA CGA AAG TC-3’
	R: 5’-GAT TTC GGC TGT TGC CAG TG-3’		R: 5’-CTG GCT GGA AGT CTC TTG CGG AG-3’
*EPOR*	F: 5’-GCG ACT TGG ACC CTC TCA TC-3’	*IL-1β*	F: 5’-CTC TGT GAC TCG TGG GAT GAT G-3’
	R: 5’-AGT TAC CCT TGT GGG TGG TG-3’		R: 5’-CAC TTG TTG GCT TAT GTT CTG TCC-3’
*Hamp*	F: 5’-GAA GGC AAG ATG GCA CTA AGC-3’	*CTGF*	F: 5’-CGT AGA CGG TAA AGC AAT GG-3’
	R: 5’-CAG AGC CGT AGT CTG TCT CG-3’		R: 5’-AGT CAA AGA AGC AGC AAA CAC-3’
*TfR2*	F: 5’-CAA GCT TCG CCC AGA AGG TA-3’	*NF-κβ*	F: 5’-ACC TGA GTC TTC TGG ACC GCT G-3’
	R: 5’-CGT GTA AGG GTC CCC AGT TC-3’		R: 5’-CCA GCC TTC TCC CAA GAG TCG T-3’
*SLC40A1*	F: 5’-CAG GCT TAG GGT CTA CTG CG-3’	*VEGF*	F: 5’-GAA GTT CAT GGA CGT CTA CCA G -3’
	R: 5’-CCG AAA GAC CCC AAA GGA CA-3’		R: 5’-CAT CTG CTA TGC TGC AGG AAG CT -3’
*HJV*	F: 5’-GCC TAC TTC CAA TCC TGC GT-3’	*Pro (III) Collagen*	F: 5’-CCA CCC TGA ACT CAA GAG TGG-3’
	R: 5’-GGT CAA GAA GAC TCG GGC AT-3’		R: 5’-CCA TCC AGA ACT GTG TAA GTG-3’
*TF*	F: 5’-GGC ATC AGA CTC CAG CAT CA-3’	*TSP-1*	F: 5’-CCG GTT TGA TCA GAG TGG T-3’
	R: 5’-GCA GGC CCA TAG GGA TGT T-3’		R: 5’ GGT TTC GGA AGG TGC AAT-3’
*Hfe*	F: 5’-CTG GAT CAG CCT CTC ACT GC-3’	*Cyt C*	F: 5’- CTT GTC ATA AAG TGG ATA TGA TC-3’
	R: 5’-GTC ACC CAT GGT TCC TCC TG-3’		R: 5’ CAA TAG GTT TGA GGC GAC ACC CTC-3’
*DMT1*	F: 5’-CAA CTC TAC CCT GGC TGT GG-3’	*HIF-2α*	F: 5’-TGA CTT CAC TCA TCC TTG CGA CCA-3’
	R: 5’-GTC ATG GTG GAG CTC TGT CC-3’		R: 5’-ATT CAT AGG CAG AGC GGC CAA GTA-3’
*TfR1*	F: 5’-GCT CGT GGA GAC TAC TTC CG-3’	*HIF-2β*	F: 5’-TGA AAG AAG GAG AAG CCC AAT A-3’
	R: 5’-GCC CCA GAA GAT GTG TCG G-3’		R: 5’-CAT CAG AGT TAT GCC GAG ACA G-3’
*TMPRSS6*	F: 5’-CCG AAT ATG AGG TGG ACC CG-3’	*18S*	F: 5’-CCA CTA AAG GGC ATC CTG GG-3’
	R: 5’-GGT TCA CGT AGC TGT AGC GG-3’		R: 5’-CAT TGA GAG CAA TGC CAG CC-3’
*BMP6*	F: 5’-GCT GCC AAC TAT TGT GAC GG-3’	*Actb*	F: 5’-GAG ATT ACT GCC CTG GCT CC-3’
	R: 5’-GGT TTG GGG ACG TAC TCG G-3’		R: 5’-CGG ACT CAT CGT ACT CCT GC-3’

### 4.6. Histopathological Analysis

Tissue samples were fixed in Bock’s fixative and embedded in paraffin wax; 4 μm thick sections were stained for routine histopathological studies with haematoxylin and eosin (H&E). Periodic acid of Shiff (PAS) was used to evaluate and confirm the levels of mesangial expansion, thickening of basement membranes and sclerotic parameters. For PAS staining, the samples were fixed in neutral formalin 10%, embedded in paraffin wax, and 4 μm thick sections were immersed in water and subsequently treated with an aqueous solution of periodic acid (1%), then washed to remove any traces of the periodic acid, and finally treated with Schiff’s reagent. All samples were examined by light microscopy using a Microscope Zeiss Mod. Axioplan 2. The degree of injury visible by light microscopy was scored by two pathologists, on a blind fashion. Lesions were evaluated on the total tissue on the slide.

Glomerular and tubulointerstitial kidney lesions were classified as mild and advanced. Mild glomerular damage was assessed by evaluating thickening of Bowman capsule, hyalinosis of the vascular pole, glomerular atrophy, hypercellularity and dilatation of Bowman´s space. Advanced glomerular damage was assessed by grading sequentially four main lesions, from least to worst one: 1—thickening of glomerular basement membrane (GBM); 2—mesangial expansion; 3—nodular sclerosis; and 4—global glomerulosclerosis. When advanced lesions were presented at a given glomerulli, the analysis of mild lesions become unavailable. Mild tubulointerstitial lesions included tubular hyaline droplets, tubular basement membrane (TBM) irregularity, tubular dilatation, interstitial inflammatory infiltration and vacuolar tubular degeneration. Advanced tubulointerstitial lesions were assessed by the presence of hyaline cylinders, tubular calcification, necrosis and the association of interstitial fibrosis and tubular atrophy (IFTA). The evaluation of vascular lesions was focused on arteriolar hyalinosis, arteriolosclerosis and arteriosclerosis. A semiquantitative rating for each slide, ranging from normal (or minimal) to severe (extensive damage), was assigned to each component, according to previously described [29]. Perl’s staining of kidney slides was performed to search for kidney iron accumulation.

### 4.7. Immunohistochemistry Analysis

Liver and renal cortex/medulla paraffin sections (4 µm) were processed for immunohistochemistry (IHC) analysis according to previously described [29]. The following primary antibodies were used: from Abcam Inc. (Cambridge, UK) for detection of hepcidin (dilution 1:150, ab81010) and CTGF (dilution 1:250, ab6992) and from Santa Cruz Biotechnology, Inc. (Santa Cruz, CA, USA) for detection of NF-κB p50 (dilution 1:500, sc-114), HIF-2α (dilution 1:250, sc-28706), and HIF-2β (dilution 1:100, sc-5581). For IHC quantification, five 400 × microscopic views of liver and renal cortex and medulla per slide were selected randomly and photographed using a Leica DFC480 microscope (Leica Microsystems, Wetzlar, Germany). The area and intensity of positive staining and the Quick Score calculation were performed according to previously described [29,74]. IHC studies were evaluated independently by two pathologists blinded to the data. Slight differences in interpretation were resolved by simultaneous viewing.

### 4.8. Statistical Analysis

For statistical analysis, we used the IBM SPSS Statistics 20 (2011). Significance level was accepted at *p* less than 0.05. Results are presented as means ± standard error of means (SEM). Comparisons between groups were performed using ANOVA and the *post hoc* Tukey test.

## 5. Conclusions

In the remnant kidney rat model of CKD-associated anemia, long-term treatment with a high rHuEPO dose is associated with development of resistance to rHuEPO therapy as a result of anti-EPO antibodies formation. In this condition, serum EPO levels are not deficient and iron availability is improved by increased duodenal absorption. The resistance to endogeneous EPO and to rHuEPO therapy seems to result from the development of a hypoxic, inflammatory and fibrotic milieu in the kidney tissue. This study provides new insights that could be important to ameliorate the current therapeutic strategies used to treat patients with CKD-associated anemia, in particular those that become resistant to rHuEPO therapy. Prevention of evolution to end-stage renal disease and increase therapy efficacy without aggravation of cardiovascular outcome is pivotal for patients suffering from CKD, which incidence is alarmingly increasing worldwide.
